# Corona – Krise – Kritik: Eine Kontroverse im Berliner Journal für Soziologie

**DOI:** 10.1007/s11609-020-00423-5

**Published:** 2020-12-08

**Authors:** Benjamin Seyd

**Affiliations:** Friedrich-Schiller-Universität Jena, Jena, Deutschland

Wenn zwei sich streiten, freut sich der Dritte, heißt es. Wenn drei sich streiten, freut sich nicht unbedingt einer der drei, womöglich aber – das jedenfalls ist die Hoffnung, die dieser Ausgabe des Berliner Journals für Soziologie zugrunde liegt – die breitere Öffentlichkeit.

Seit Beginn der großen Pandemie stehen wir als wissenschaftliche Zeitschrift vor einem Dilemma. Einerseits ist „Corona“ als genuin soziologisches Thema nicht zu ignorieren – zu Recht wird darauf hingewiesen, dass die Stunde der Soziologie gerade in Krisen wie dieser schlägt und sie in Krisen ihre „Systemrelevanz“ (Becker 2020) bekundet. Andererseits ist es nichts Neues, dass diejenigen, denen die Stunde schlägt, nicht immer bereit sind: Wissenschaftliche Erkenntnis braucht ihre Zeit. In einem frühen lesenswerten Beitrag zur Krise notierte Branko Milanovic^1^:

[W]ir haben keine Ahnung, wie lang die Pandemie andauern wird, wie viele Länder sie treffen wird, wie viele Menschen sterben werden, ob der gesellschaftliche Zusammenhalt zerreißt oder nicht. Wir tappen völlig im Dunkeln. Das meiste, was wir heute sagen, kann sich morgen schon als falsch erweisen. Wenn jemand mit seinen Prognosen richtig liegt, dann muss das nicht heißen, dass er schlau ist, sondern er kann einfach Glück haben. Doch in solch einer Krise macht Glück viel aus...

Nun – inzwischen wissen wir deutlich mehr. Wir wissen, dass die Pandemie kein einziges Land verschont hat, dass sie – Stand 25. November 2020 – weltweit über 59 Mio. Menschen infiziert hat und für mindestens 1,4 Mio. Todesfälle verantwortlich ist.^2^ Und wir können sehr viel mehr sagen über die Belastungen, Begünstigungen und Bewältigungsversuche, die sie mit sich gebracht hat. Wir wissen auch sehr viel mehr über das Virus selbst – nicht zuletzt, dass es sich nicht so einfach bezwingen lässt, wie manch einer, sogar manch ein gescheiterter Staatenlenker, dachte. Das gilt auch für Europa und Deutschland insbesondere, das nach einem Sommer voller Zuversicht am Anfang eines langen Winters steht. Das heißt auch: Die Dinge sind noch immer im Fluss, die Volatilität aller Einschätzungen hält noch immer an und spiegelt sich nicht zuletzt auch in der schwankenden Qualität der immer zahlreicheren sozialwissenschaftlichen Deutungsversuche.^3^

Doch dürfte sein Glück zu sehr strapazieren, wer mit dem Denken zu spät anfängt. Damit aber wird die Lösung des Dilemmas – Reden ist heikel, Schweigen ist nichts – zu einer Frage nach der richtigen Form. Auf diese Frage versuchen wir, mit der vorliegenden Kontroverse, die zugleich eine neue Rubrik im Berliner Journal inauguriert, eine Antwort zu geben.

Zwei Absichten sind mit dieser Antwort verbunden: Zum einen soll der – kollegiale, aber zuweilen auch leidenschaftlich und mit Vehemenz geführte – Austausch dreier Sozialwissenschaftler, die sich beileibe nicht in allem einig sind, die strittigen Fragen, um die es zu ringen gilt, *als solche* transparent machen. Anders als die Virologie formiert sich die Soziologie nicht um einen Bestand positiven Wissens, sondern spürt im unüberschaubaren Geflecht sozialer Bezüge Zusammenhängen nach, um die es sich zu streiten lohnt; und zielt dabei weniger auf abschließende Erkenntnisse als auf „Orientierungswissen“ (Müller 2018, S. 474), mit dem sich etwas anfangen lässt. Schon dem Namen nach kann eine Corona-*Kontroverse* nicht einfach sagen, was Sache ist in Sachen Corona. Aber sie kann deutlich machen, worauf es ankommt: auf welche Fragen, auf welche möglichen Antworten, auf welche Argumente.

Zum anderen war uns wichtig, „Corona“ in einen größeren Zusammenhang einzuordnen. Corona ist sicher keine neue „Stunde Null“, wie verschiedentlich behauptet wird. Im Naturzustand Hobbes’scher Prägung mag das Leben immer schon „einsam, armselig, scheußlich, tierisch und kurz“ sein, unter solchen Umständen wäre eine Pandemie allerdings kaum mehr der Rede wert. SARS-CoV‑2 entfaltet seine Wirkung jedoch unter den sehr spezifischen Bedingungen moderner, globaler, komplexer und nicht zuletzt überwiegend kapitalistischer Gesellschaften. Diese Bedingungen gilt es zu reflektieren, wenn Gehaltvolles über seine gesellschaftliche Bedeutung herausgefunden werden soll.

Klaus Dörre, Hartmut Rosa und Stephan Lessenich können für diese Einordnung der Corona-Krise durchaus als prädestiniert gelten; jedenfalls fangen sie ihre Debatte nicht bei Null an. Krisen- wie kontroversenerprobt, haben sie 2009 bereits das Debattenbuch *Soziologie – Kapitalismus – Kritik* vorgelegt, in dem sie die Finanzkrise und ihre Verwerfungen am paradigmatischen Leitfaden der „Landnahme“ (Dörre), „Aktivierung“ (Lessenich) und „Beschleunigung“ (Rosa) deuten. Danach haben Sie ab 2011 im Rahmen des Jenaer DFG-Kollegs „Postwachstumsgesellschaften“ im doppelten Sinne miteinander weitergestritten – nun mit verstärkter Aufmerksamkeit v. a. für die ökologische Dimension der Krise. Es ging um die „ökonomisch-ökologische Zangenkrise“ (Dörre), die die „dynamische Stabilisierung“ (Rosa) in den nördlichen „Externalisierungsgesellschaften“ (Lessenich) unter Druck setzt. Eine „konstruktive Kontroverse“, die im vorigen Jahr mit der Frage (und dem Kongress) nach der „Großen Transformation“ und zur „Zukunft moderner Gesellschaften“ ihren vorläufigen Abschluss fand.

Damit sind sie zugleich gerüstet, eine Beobachtung aufzunehmen, die oft gemacht, aber vielleicht dennoch nicht ausreichend reflektiert wurde: dass nämlich die Pandemie als eine vermeintliche „Naturkatastrophe“ in vielerlei Hinsicht einer ähnlichen Logik folgt wie jene längerwährende Entwicklung, die sie vom Spitzenplatz des öffentlichen Krisenbewusstseins verdrängt hat. Wie die ökologische Krise und insbesondere der Klimawandel entspringt die Pandemie einer Umwelt, die aufs Engste mit den menschlich-modernen Bestrebungen verwoben ist und sich der Ausdehnungsbewegung der Menschheit und ihrer Produktivkräfte doch nicht ohne Weiteres fügt. Hier wie da stellt sich die Frage nach der richtigen Abwägung von Wirtschaft und (viraler) Ökologie; und hier wie dort findet diese Abwägung ihre äußerste Grenze in der vollständigen Leugnung des Problems, von Fall zu Fall noch unterfüttert durch verschwörungstheoretische Behelfsrationalisierungen, die ihre ganz eigene Ansteckungswirkung entfalten. Wenn Klaus Dörre die Pandemie mit Fernand Braudel als einen „äußeren Stoß“ bezeichnet, Stephan Lessenich dies mit den Mitteln seines Externalisierungsansatzes hinterfragt und Hartmut Rosa auf die messbare, sogar mit geminderter seismischer Aktivität einhergehende Stillstellung gesellschaftlicher Vorgänge verweist, dann geht es um die Frage, wie die Corona-Krise überhaupt zu verstehen ist. Ist sie ein unvorhersehbares Unglück, ein sogenannter schwarzer Schwan? Oder vielmehr ein „schwarzer Elefant“^4^, eine offensichtliche Bedrohung, die abzusehen war, aber ignoriert wurde? Ist sie gar, wie Bob Dylan mehr geraunt denn behauptet hat, „ein Vorbote für etwas anderes, das auf uns zukommt“ (Brinkley 2020)? Die Frage nach der *„Natur“ der Krise *ist die *erste von vier Leitfragen*, die die Debatte strukturieren.

Diese erste ist aufs engste mit einer *zweiten Leitfrage *verbunden: Fragt jene nach der Intension der „Corona-Krise“, betrifft diese ihre Extension, ihre *Ausdehnung*. Viel wurde im Frühjahr darüber gerätselt, welche Art von Aufschwung dem ökonomischen Einbruch wohl folgen würde: Wird es ein V? Oder ein U? Ein W? Eine umgekehrte Quadratwurzel, ein „Nike-Swoosh“, gar ein K? Oder, der apokalyptischste unter den Buchstaben, ein L? Dahinter steckt die Frage, ob die Krise eine konjunkturelle oder vielmehr eine strukturelle ist — und wenn ja, in welcher Weise und in welchem Ausmaß. Lässt sich sagen, dass die präpandemische Lage eigentlich ganz in Ordnung war? Ist COVID-19 selbst die Krise und also vorbei, sobald die Pandemie überwunden, die „Herde“ geimpft oder immun ist? Oder irren die, die dies behaupten, wie einst Herbert Hoover, als er angesichts der Großen Depression verkündete, das „grundlegende Geschäft“ der Vereinigten Staaten stehe „auf stabilen und gesunden Füßen“? Unsere drei Referenten hegen keinen Zweifel daran, dass SARS-CoV‑2 eine fundamentalere Unwucht zum Tragen bringt, die weit über das Infektionsgeschehen hinausreicht. Das allein ist natürlich längst keine besonders steile These mehr, braucht es doch kaum mehr ein seismographisches Instrumentarium, um die Gegenwart als von einem „Wust von Krisen“^5^ gezeichnet zu erkennen. Doch selbst wenn man sich darauf einigt, kann das Strukturelle der Krise sich auf Verschiedenes beziehen: Ist die Pandemie die *Ursache* krisenhafter Kaskadeneffekte? Ist sie *Verstärker* einer ohnedies brisanten Lage? Oder *Katalysator* eines vorhandenen Krisen*potenzials*?

Der enge Ausgang der US-amerikanischen Präsidentenwahl sollte vielleicht als letzte Mahnung dienen, nicht zu sehr darauf zu hoffen, dass es sich bei den Wirrungen dieser Tage nur um vorübergehende Aberrationen handelt. Offensichtlich haben sich Stagnation, Ungleichheit und ökologische Zumutungen unter den Bedingungen der Globalisierung zu einem explosiven Gemisch verbunden, das zunehmend politische Wirksamkeit entfaltet. Was genau diese Wirksamkeit bedeutet und was genau das Gewebe von Bedingungen ausmacht, auf die die Pandemie trifft, treibt unsere drei Autoren in ihren Debattenbeiträgen um. Einigkeit besteht zwischen ihnen dahingehend, dass es bei „Corona“ nicht nur um Corona geht, sondern die Krise tiefer reicht. Doch gibt es Gradierungen der Zwangsläufigkeit: Ist die Pandemie für Stephan Lessenich eine immanente Folge der Verdrängungslogik nördlicher Wohlstandsgesellschaften, gesteht Klaus Dörre ihr als „externer Schock“ ein stärker kontingentes Moment zu. Gleichwohl mache sie „wie durch ein Brennglas“ die Anfälligkeiten einer Globalisierung sichtbar, die „repulsiv“ zu werden droht und neben Ökonomie und Ökologie auch längst die Demokratie in Mitleidenschaft gezogen hat. Anfälligkeiten einer auf permanente Steigerung angelegten Gesellschaftsformation beobachtet auch Hartmut Rosa, betont aber ein stärker politisches Moment, insofern erst durch Interventionen des Staates die Bremsen am Räderwerk der Beschleunigung angelegt werden.

Diese Unterschiede gehen einher mit unterschiedlichen Haltungen in der *dritten Leitfrage* nach den *Konsequenzen* der Pandemie. Ein frühes Interview von Hartmut Rosa, in der er die „Chance“ betonte, die in der Krise stecke, gab den Anlass für die hier vorliegende Debatte. Dabei geht es Rosa nicht darum, der Krankheit ihren Schrecken abzusprechen, er findet jedoch Grund für Zuversicht darin, dass politische Entscheidungen der Logik der (Profit‑)Maximierung aktiv Einhalt gebieten – und so zeigten, dass genau das möglich sei. Dagegen blicken Stephan Lessenich und Klaus Dörre skeptischer auf die Effekte der Pandemie auf individuelle wie kollektive Handlungsspielräume. Die Frage ist dann: Chancen welcher Art, wo, für wen? Wer profitiert, und wer nicht? Und trägt diese Konstellation in der Tat den Keim zur Trendumkehr in sich? Oder gilt das allenfalls für die, die schon vorher auf der sozialversicherungspflichtigen Sonnenseite der Homeoffice-Befähigten standen? Ist die Krise ein Ausweis zwischenmenschlicher Solidarität, oder geht es von vornherein nur um die Solidarität jener, die die Gnade der nördlichen Geburt erfahren haben? Keiner der drei Autoren ist ohne Hoffnung – doch in ihrer Qualität wie ihrer Intensität ist die Hoffnung jeweils ganz unterschiedlich gelagert.

Dabei gilt: Wo Chancen zu nutzen und Gefahren abzuwenden sind, da geht es – wie Hartmut Rosas titelgebender Hinweis auf die „Bifurkationspunkte“ verdeutlicht – um politisches Handeln. Allerdings gibt es kaum ein treffenderes Beispiel dafür, dass es auf Interpretationen ankommt, wo die Welt verändert und die Zukunft gestaltet werden soll, als die Corona-Krise. Das gilt für schlechte Deutungen wie für gute, und nebenbei auch für die Frage, was die einen von den anderen unterscheidet. Es gilt für Verschwörungstheorien wie für virologische Expertisen, auch wenn deren Wahrheitswert jeweils ein anderer ist. Und es gilt nicht zuletzt für die Soziologie: Welche Rolle kommt ihr zu in diesem Wettbewerb um eine passende Sicht auf die Dinge? Und auch wenn sie keine end-gültigen Antworten parat hat: Was kann sie selbst zu einem guten Ende der Krise beitragen? Diese Frage nach der *Aufgabe der Soziologie* ist die *vierte und letzte Leitfrage*, die und der sich unsere drei Diskutanten gestellt haben. „Best account“, „Wahrheitspolitik“ und „Public Sociology“ sind die Stichwörter, um die sich ihre Antworten sortieren.

Jeweils wird deutlich: Es kann nur darum gehen, Angebote zu machen, eine Einladung zum Weiterdenken, zum Nachfragen, zum Widerspruch. Und genau so ist die neue Rubrik „Debatte“ gemeint, der wir von hier an ein langes und aufregendes Leben wünschen. Wir würden uns freuen, wenn andere die Gedanken fortspinnen und die Corona-Kontroverse über die ersten drei Texte hinaus eine Fortsetzung fände. Zugleich sollen unter dem neuen Dach auch andere wichtige Fragen ihren Platz finden – schließlich besteht das Krisen-Knäuel, mit dem Soziolog_innen sich vorzugsweise abmühen, aus ganz verschiedenen Fäden, die sich aufnehmen lassen. Nicht mehr als ein Anfang ist gemacht – und in diesem Sinne hoffen wir auf (Ihre) Fortsetzung.

Derweil gehört zu einem konstruktiven Umgang mit der Pandemie, ihr unsere Aufmerksamkeit zuzuwenden, aber nicht komplett unterzuordnen. Die weiteren Abhandlungen in diesem Heft bieten daher ungerührt von kurzfristigen Diskursverschiebungen Einblicke in die laufende soziologische Forschungsarbeit. An eher unterschwelligen begrifflichen Veränderungen interessiert sind die theoretischen Beiträge von Lars Gertenbach und Susann Wagenknecht. Ersterer widmet sich der Systematisierung der unübersichtlich gewordenen Debatte um Performativität – ein Konzept, das in Zeiten unzuverlässiger Faktizität fraglos große Erklärungskraft verspricht, in seiner „performance“ dabei aber selbst nicht immer stabil ist. Gertenbach folgt den Wendungen der performativen Wende von John L. Austin über Pierre Bourdieu und Judith Butler bis zu Karen Barad und Michel Callon und gelangt bei seiner Neuvermessung des Feldes zu unerwarteten Brückenschlägen und überraschenden Grenzziehungen. Die Kartografie, die er schließlich vorlegt, unterscheidet zwei Gebiete: ein sprachtheoretisches, das Performativität in Verlängerung des Linguistic Turn versteht, und ein medientheoretisches, das über ihn hinausgeht. Sicher ist eine gute Karte keine Garantie dafür, dass man sein Ziel am Ende erreicht; doch eine gute Voraussetzung dafür ist sie auf jeden Fall.

Ebenfalls in theoretischen Gefilden unterwegs ist Susann Wagenknecht in ihrem Aufsatz, aber in den anderen (oder angrenzenden) der Praxistheorie. Darin macht sie sich auf die Suche nach dem, was Praktiken im Innersten zusammenhält – und folgt dabei der Spur der Normativität. Mag das zunächst wie eine Rückkehr zum Obrigkeitsdenken früherer soziologischer Handlungstheorien erscheinen, das Handeln als durch Normen bestimmt verstand, bleibt Wagenknecht mit beiden Beinen auf dem Boden der Praxistheorie: Die Regeln, die sie für des Rätsels Lösung ins Auge fasst, sind ihr ein fesselnder Untersuchungsgegenstand, aber sie fesseln nicht das Handeln der Subjekte. Vielmehr werden sie im Handeln mobilisiert und dabei – wie Wagenknecht mit Joseph Rouse und Theodore Schatzki rekonstruiert – zugleich interpretiert und konstituiert. Auf dem praxistheoretischen Gelände entspinnt sich somit ein Netzwerk sich wechselseitig „ver-antwortender Bezüge“, das u. a. einen soziologischen Klassiker wie Popitz mit Experten für Ampelschaltungen, dem Kinderliedermacher Rolf Zuckowski und ausrastenden Autofahrern auf L.A.s Straßen in Verbindung bringt. Wagenknecht hat selbst ein Talent dafür, solche Bezüge herzustellen, die zu kartografieren allerdings alles andere als einfach ist: Denn die Kartografin trägt mit ihren Bezugnahmen selbst zur Landschaft bei – auch die praxistheoretische Beschreibung der Wirklichkeit hat etwas Performatives.

Etwas kleinteiliger, aber mit großer Sorgfalt, widmet sich schließlich Markus Lörz in seinem Aufsatz der titelgebenden Frage: „Warum nehmen Männer mit Migrationshintergrund überproportional häufig ein Studium auf, gelangen aber am Ende seltener in die weiterführenden Masterstudiengänge?“ Wer von Wissenschaft, zumal von soziologischer Forschung erhofft, dem stahlharten Gehäuse der Moderne mit stahlharten Antworten beizukommen, wird sich mit Lörz’ bedachtem Vorgehen möglicherweise schwertun; allen anderen könnte das Aufspüren und Kleinarbeiten der Widersprüche und das tentative Abwägen von Erklärungsangeboten dagegen als Muster wissenschaftlicher Redlichkeit dienen. Zwar kann Lörz mithilfe einer Regressionsanalyse die Umkehr des Interaktionseffekts von Geschlecht und Migrationshintergrund während des Studienverlaufes nachweisen, doch an der Erklärung von dessen Ursachen beißen sich die verfügbaren Erklärungsmodelle die Zähne aus. Auch das hat etwas von stählerner Härte, aber es ist eher eine harte Frage – oder die Frage einer harten Wirklichkeit, die sich als „deutlich komplexer als theoretisch angenommen“ erweist –, mit der Lörz die empirische Bildungsforschung konfrontiert.

Man kann festhalten: Akribische Wissenschaft ist mühsam, doch sie ist, wie sich immer wieder zeigt, die einzige, die die Mühe wirklich wert ist. Garantien, dass der Aufwand sich am Ende auszahlt, gibt es natürlich nicht. Doch hält er in diesen Zeiten coronabedingter wie -unbedingter Unsicherheit unsere Hoffnung aufrecht, dass das Leben nach Corona weder (im Sinne des Orwell’schen Esels aus *Farm der Tiere*) weitergehen wird wie immer („das heißt, schlecht“), noch die Welt gar, wie Michel Houllebecq (2020) in Abwandlung des tierischen Bonmots prophezeit, „dieselbe sein [wird], nur in etwas schlimmer“. Zwar werden wir wahrscheinlich auch „nach dieser Ausgangssperre nicht in einer neuen Welt aufwachen“ (ebd.). Und sicher – es gehört Glück dazu. Doch vielleicht – vielleicht! – ist es ja das Glück des Tüchtigen, das am Ende den Unterschied ausmacht.

## Footnotes

1 Erschienen in Englisch auf Milanovic’ Blog (2020, Übersetzung B.S.).

2 Daten laut dem WHO Coronavirus Disease (COVID-19) Dashboard, https://covid19.who.int/. Zugegriffen: 26.11.2020.

3 Für den bis dato vielleicht umfassendsten Überblick über die Diskussion siehe Volkmer und Werner (2020).

4 Der Begriff „black elephant“, eine metaphorische Kreuzung aus dem unerwartbaren „black swan“ und dem offensichtlich-unausgesprochenen „elephant in the room“, wurde von dem Umweltschützer Adam Sweidan für den absehbaren Zusammenbruch der marinen Ökosysteme geprägt und von Thomas L. Friedman im Zusammenhang der Corona-Pandemie aufgegriffen (Friedman 2020).

5 So das Editorial Board der New York Times (2020) in seinem Kommentar in Reaktion auf den Wahlsieg von Joe Biden bei der US-Präsidentenwahl, das weiter konstatiert, „kürzliche Ereignisse hätten viele der bestehenden Vorerkrankungen offengelegt und oftmals verschärft“.
